# Cost-effective medium optimization and functional enhancement of *Lactiplantibacillus plantarum* 22F for industrial-scale probiotic production in swine feed

**DOI:** 10.14202/vetworld.2025.1759-1776

**Published:** 2025-06-27

**Authors:** Nay Zin Myo, Ratchnida Kamwa, Benjamas Khurajog, Pawiya Pupa, Wandee Sirichokchatchawan, David J. Hampson, Nuvee Prapasarakul

**Affiliations:** 1 Department of Microbiology, Faculty of Veterinary Science, Chulalongkorn University, Bangkok 10330, Thailand; 2 The International Graduate Course of Veterinary Science and Technology, Faculty of Veterinary Science, Chulalongkorn University, Bangkok 10330, Thailand; 3 Department of Biology, Faculty of Science, Chulalongkorn University, Bangkok 10330, Thailand; 4 Center of Excellence in Diagnosis and Monitoring Animal Pathogens, Faculty of Veterinary Science, Chulalongkorn University, Bangkok 10330, Thailand; 5 College of Public Health Sciences, Chulalongkorn University, Bangkok 10330, Thailand; 6 School of Veterinary Medicine, Murdoch University, Perth, Western Australia, 6150, Australia

**Keywords:** cost-effective production, fermentation optimization, *Lactiplantibacillus plantarum* 22F, metabolomics, modified medium, stress tolerance, swine probiotic

## Abstract

**Background and Aim:**

Industrial-scale probiotic production requires economically viable media formulations that do not compromise strain functionality. This study aimed to develop a cost-effective medium for cultivating *Lactiplantibacillus plantarum* 22F (L22F), a probiotic candidate isolated from swine feces, while evaluating its industrial viability and functional metabolic profile.

**Materials and Methods:**

Carbon (glucose, sucrose, and dextrose) and nitrogen (yeast extract, soy protein isolate, and whey protein concentrate) sources were screened using one-variable-at-a-time and Plackett–Burman design, followed by Response Surface Methodology for optimization. Fermentation was scaled from a flask to 50 L fermenters at 37 °C and pH 6.50 ± 0.05. Cell viability, pH, and residual sugar were monitored. Functional assessments included stress tolerance assays (heat, acid, bile, and oxidative stress) and untargeted metabolomic profiling using ultra-high performance liquid chromatography-quadrupole time-of-flight mass spectrometry.

**Results:**

The optimal medium comprised 9 g/L glucose, 14.1 g/L soy protein isolate, and 14.1 g/L yeast extract, supplemented with minerals. In 50 L fermentation, L22F achieved 9.20 log colony-forming units/mL at 12 h, with residual sugar at 1.50 g/L and pH 3.99. Compared to de Man, Rogosa, and Sharpe, the modified medium reduced production cost by 70%–88%, improved fermentation efficiency, and supported enhanced stress resilience. Metabolomic analysis revealed an elevated production of bioactive metabolites, particularly 1,4-dihydroxy-2-naphthoic acid and indolelactic acid, which are known to support gut homeostasis, anti-inflammatory responses, and probiotic efficacy.

**Conclusion:**

This study presents a cost-effective and scalable fermentation medium specifically designed for high-density L22F production. Beyond economic advantages, the medium enhanced the functional properties of L22F, supporting its application as a sustainable probiotic feed additive for swine. These findings establish a foundation for further industrial application and *in vivo* validation.

## INTRODUCTION

Probiotics, especially lactic acid bacteria (LAB), have garnered considerable interest as additives in food and feed owing to their recognized health-promoting properties. These beneficial microorganisms play a crucial role in supporting gastrointestinal health, enhancing immune function, and contributing to overall well-being [1]. Although numerous microorganisms are employed as dietary supplements, *lactobacilli* are among the most widely commercialized probiotics globally. Of the over 50 known Lactobacillus species, *Lactiplantibacillus plantarum* – formerly known as *Lacto-bacillus plantarum* – is notably versatile and commonly utilized in the fermentation of vegetables, meats, and dairy products [2]. Various strains of *L. plantarum* have demonstrated the ability to synthesize antimicrobial compounds and inhibit pathogenic and spoilage organisms [3]. These bioactivities underline the species’ potential application as a functional ingredient in the food industry [4].

In the previous study by Sirichokchatchawan *et al*. [5], multiple probiotic strains were isolated from swine fecal samples, among which *L. plantarum* strain 22F (L22F) exhibited superior probiotic traits and conformed to the European Food Safety Aut-hority criteria by lacking antimicrobial resistance genes. In addition, L22F displayed potent *in vitro* antibac-terial, antiviral, anticonjugation, and antibiofilm activities [6–8]. We also developed a double-layer microencapsulation technique suitable for field application in pig farms, which effectively enhanced gut health and growth performance in swine throughout the rearing phase. Building on these findings, Pupa *et al*. [9–11] recommended the integration of L22F as a feed additive in commercial swine production through an industrially scalable microencapsulation approach.

As the demand for probiotic-enriched food and feed products continues to grow, economically sustain-able industrial production methods are increasingly essential. Key components of probiotic manufacturing include strain selection, fermentation, and downstream processing, with the fermentation medium constituting approximately 30%–40% of total production costs [12]. Although commercially available media such as de Man, Rogosa, and Sharpe (MRS) are effective, they are often prohibitively expensive due to their complex and costly formulations [13]. Moreover, such media may not always provide optimal growth conditions for all probiotic strains. To address these limitations, there has been a growing interest in developing modified media that are both cost-effective and nutritionally tailored to the specific needs of LAB strains [14–17].

Formulating economical media for large-scale LAB production generally entails the optimization of nutrient composition, fermentation parameters, and process conditions. Various statistical tools, including the One-Variable-at-a-Time (OVAT) method, Plackett–Burman Design (PBD), Response Surface Methodology (RSM), and factorial designs, are employed to achieve these goals. In the present study, the OVAT method was initially employed for its straightforward application in single-factor screening. Subsequently, PBD was used to efficiently identify significant variables among a larger set with fewer experimental runs. Finally, RSM was applied to refine and optimize the concentrations and interactions of the selected variables. This multistep approach ensures a cost-efficient, systematic, and statistically robust strategy for optimizing media for industrial-scale LAB cultivation [18–20]. We previously employed this methodology to develop media for the industrial-scale propagation of *Pediococcus acidilactici* 72N [21]. Common modifications include replacing high-cost ingredients with more affordable alternatives, fine-tuning nutrient ratios to meet strain-specific requirements, and adjusting fermentation parameters to enhance productivity. Nitrogen sources derived from the food industry by-products – such as whey protein, soy protein, yeast extract, and meat extract – have shown promise as economical substitutes in media formu-lation [13, 22].

Similarly, the choice and management of carbon sources play a pivotal role in optimizing microbial growth and product yield during fermentation. In this study, glucose, dextrose monohydrate, and sucrose, both affordable and widely available in Thailand, were selected as carbon sources for screening. Tailoring car-bon source concentrations to suit the metabolic preferences of specific strains is critical to minimizing residual sugars and maximizing fermentation efficiency. Moreover, fermentation conditions such as temperature, pH, and agitation speed must be optimized to achieve high biomass yields and consistent product quality. Through meticulous formulation and process adjustments, higher viable LAB cell counts can be attained while substantially reducing production costs.

Furthermore, the composition of the growth medium has been shown to significantly affect key probiotic properties, including bile salt resistance, acid tolerance, and the synthesis of health-promoting meta-bolites [23–26].

Despite the growing interest in LAB as probiotics for animal feed applications, the industrial-scale production of these organisms remains constrained by the high cost of fermentation media and a lack of formulations tailored to strain-specific requirements. Most commercially available media, such as MRS, are designed for general LAB cultivation and do not account for cost efficiency or the metabolic and functional characteristics of specific strains such as L22F. Although several studies have investigated low-cost media alternatives, they have predominantly emphasized cell yield rather than maintaining or enhancing functional probiotic attributes, such as stress tolerance, metabolite biosynthesis, and antagonistic activity. Moreover, the impact of medium composition on the metabolomic profile of probiotic strains remains underexplored, particularly with respect to bioactive compounds that support gut health and immunomodulation. There is a critical need for integrated approaches that combine cost-effective medium optimization with comprehensive assessments of strain functionality under industrial conditions.

The primary aim of this study was to develop a cost-effective and scalable fermentation medium optimized for the industrial cultivation of L22F, a swine-derived probiotic candidate with demonstrated antibacterial and immunomodulatory properties. To achieve this, we employed a stepwise optimization strategy that combined OVAT, PBD, and RSM to identify the most suitable food-grade carbon and nitrogen sources. In addition, we sought to evaluate the impact of the optimized medium on L22F’s viability, stress tolerance (heat, acid, bile, and oxidative), and metabolomic profile through untargeted liquid chromatography–mass spec-trometry analysis. By addressing both economic and functional parameters, this study aims to establish a robust platform for the cost-efficient industrial prod-uction of L22F as a feed additive in swine farming.

## MATERIALS AND METHODS

### Ethical approval

The experimental protocol (Protocol No. IBC-2431023) was approved by the Institutional Biosafety Committee of the Faculty of Veterinary Science, Chulal-ongkorn University.

### Study period and location

The study was conducted from June 2022 to May 2024. All experiments were conducted at the Department of Microbiology, Chulalongkorn University, Bangkok, except for the scale-up fermentation experiment, which was conducted at K.M.P. Biotechnology Co., Ltd., Chonburi.

### Probiotic strain and culture conditions

L22F used in this study was isolated from the feces of a Thai commercial pig and was suggested as a pot-ential probiotic candidate for use as a feed additive [5]. The strain was stored at −20°C in MRS broth (Difco, USA) containing 20% glycerol. Following thawing, the res-ulting mixture was subcultured on an MRS agar plate and incubated at 37°C for 48 h for subsequent use [7].

### Assessment of carbohydrate use

Carbohydrate use by strain L22F was assessed using the Automated VITEK^®^ 2 compact system (bioM-érieux, France). This system was selected for the study due to its faster turnaround time (typically within 6–8 h), reduced human error, and objective result interpretation. The sample preparation procedure was performed according to the manufacturer’s instructions. Strain L22F was subcultured on an MRS agar plate and incubated at 37°C for 48 h to ensure optimal colony morphology and viability. Subsequently, 1–2 colonies from the freshly cultured plate were selected and transferred into a 3 mL tube containing a 4.5% NaCl solution. The cell suspension was vortexed and its density was checked to ensure a range of 0.5–0.63 using McFarland standards. The VITEK^®^ 2 Colorimetric Bacterial Card identification card was inserted into the prepared L22F cell suspension tube and then loaded into the machine for analysis.

### Preparation of bacterial cell suspensions

From the freshly cultured MRS plates, colonies of L22F were transferred into 10 mL of MRS broth and incubated at 37°C for 18 h under static conditions. The culture was then centrifuged at 2,795 × *g* at 4°C for 10 min. The supernatant was removed, and the cell pellets were washed twice with 0.85% saline solution. Cells were resuspended in saline, and the optical density at 600 nm was adjusted to achieve a concentration of approximately 10^8^ colony-forming units per milliliter (CFU/mL).

### Laboratory-grade and food-grade media components, minerals, and buffering agents

Laboratory-grade and food-grade components were used for microbial cultivation and media optim-ization, respectively, and were selected based on their purity and suitability for the intended application scale. Commercial MRS broth and agar (Difco) were used as the standard medium for cell culture and enumeration. Laboratory-grade minerals and buffering agents, including Tween 80 (Q RëC™, New Zealand), ammonium citrate (C_6_H_14_N_2_O_7_) (Sigma-Aldrich, Germ-any), di-potassium hydrogen phosphate (K_2_HPO_4_) (Carlo Erba, Italy), sodium acetate (CH_3_COONa) (Carlo Erba), magnesium sulfate heptahydrate (MgSO_4_·7H_2_O) (Fluka™, Germany), and manganese sulfate monohyd-rate (MnSO_4_·H_2_O), were incorporated into all formul-ations in controlled experiments (from 10 mL to 500 mL experiments). For cost-effective and scalable formulation development, food-grade ingredients were sourced locally from suppliers in Bangkok, Thail-and. Carbon sources, such as glucose and dextrose monohydrate, were obtained from Krungthepchemi Company (Bangkok, Thailand), whereas sucrose was obtained from Mitr Phol Company (Bangkok, Thailand). Nitrogen sources included yeast extract from Mighty Company (Bangkok, Thailand), soy protein isolate, and whey protein concentrate (WPC) from Krungthepchemi. In addition, food-grade minerals and buffering age-nts – Tween 80 and MgSO_4_·7H_2_O (Krungthepchemi), CH_3_COONa and MnSO_4_·H_2_O (Chemrich, Thailand), and K_2_HPO_4_ (Thepthai Chemical Company, Thailand) – were used in place of laboratory-grade minerals and buffering agents in 5 L and 50 L experiments.

### Hydrolysis of WPC and soy isolate protein (SIP)

The hydrolysis of WPC and SIP was performed before each experiment to enhance the protein properties. WPC and SIP were reconstituted in distil-led water to obtain a solution with the required conce-ntration for each trial, as follows: 15 g/L and 30 g/L of both WPC and SIP for the PBD; 10, 14.1, 20, 26, and 30 g/L of SIP for the RSM; and 14.1 g/L of SIP for the flasks, bioreactors, and fermenters experiments. Following our modified technique, based on that described by Galante *et al*. [10], the solutions were subjected to acidic hydrolysis with 1 M hydrochloric acid until a pH of 4.0 was reached and then heated at 100°C for 30 min. These hydrolysis parameters were selected to enable efficient cleavage of labile bonds, such as glycosidic or peptide linkages, in WPC and SIP while minimizing the degradation of sensitive bioactive compounds by balancing acidity, heat, and exposure time. The hydrolysate was cooled to room temperature and then filtered using a polypropylene filtration cloth to remove the solids. The filtered solutions were used for optimization and scale-up experiments.

### Measurement parameters

The drop plate technique was used for cell enumeration in all media optimization and scale-up experiments. The viable cell numbers were determined using MRS agar. The pH values and residual sugar contents were determined in all scale-up experiments. The pH values were measured using a pH meter (METTLER-TOLEDO, Thailand). The residual sugars were assessed using the 3,5-dinitrosalicylic acid method [27]. In this procedure, 1 mL of each sample or glucose standard was mixed with 1 mL of dinitrosalicylic acid (DNS) reagent and heated in a boiling water bath for 5–10 min. After cooling, 1 mL of sodium potassium tartrate was added to stabilize and intensify the color. The absorbance was measured at 540 nm, and the reducing sugar concentrations were calculated using a glucose standard curve.

### Media optimization

#### Screening of carbon sources

The OVAT approach was employed to investigate how various carbon sources impacted the growth of the L22F strain. Various food-grade carbon sources, including glucose, dextrose, and sucrose, were tested at concentrations of 10, 20, and 30 g/L, each combined with 10 g/L of yeast extract. A mixture of laboratory-grade minerals and buffering agents, consisting of 1 g/L Tween 80, 2 g/L C_6_H_14_N_2_O_7_, 2 g/L K_2_HPO_4_, 5 g/L CH_3_COONa, 0.1 g/L MgSO_4_·7H_2_O, and 0.05 g/L MnSO_4_·H_2_O, was added to each medium formulation. The initial pH of all media was adjusted to 6.5 ± 0.05 using 1 M sodium hydroxide (NaOH) and 1 M hydrochloric acid (HCl) and sterilized at 121°C for 20 min. The experiment was conducted in 15 mL screw-cap tubes containing 10 mL of medium, inoculated with 1% (v/v) inoculum (100 μL) from the cell suspension (~10^8^ CFU/mL) of strain L22F. After incubating at 37°C for 24 h under static conditions, viable cell counts were measured as described in the “Measurement Parameters” section. Experiments were conducted in triplicate and the carbon source that most significantly increased viable cell production was selected for subsequent studies.

#### Screening of nitrogen sources

The PBD was used to identify key nitrogen sources that affect viable cell production in strain L22F. This design included a mixture of three nitrogen sources: WPC, SIP, and yeast extract. The concentration levels of these nitrogen sources were determined using Plackett–Burman statistics, resulting in a design matrix with experimental runs and coded values (+1, −1) representing different concentrations. The media were prepared according to these combinations, incorporating the optimal carbon source for L22F identified in a previous experiment. The same concentrations of laboratory-grade minerals and buffering agents were added to each formulation, and the initial pH of all media was adjusted to 6.5 ± 0.05 using 1 M NaOH and 1 M HCl. The media were then sterilized at 121°C for 20 min. After sterilization, 1% (v/v) inoculum (100 μL) from the L22F cell suspension (~10^8^ CFU/mL) was added to 10 mL of each medium formulation, followed by incubation at 37°C for 24 h under static conditions. Viable cell counts were determined as described in the “Measurement Parameters” section, with all experiments conducted in triplicate. Nitrogen sources significantly enhancing viable cell production (confidence level >95%) were selected for further optimization studies.

#### Optimization of media component concentrations using the RSM

The RSM was applied based on Central Composite Design (CCD) to optimize the concentrations of me-dium components and to evaluate the effects of each variable and their interactions. Based on the results of the PBD experiment, appropriate carbon and nitrogen sou-rces were selected as independent variables in this study. After determining the concentration levels of these variables, CCD provided a design matrix with experimental runs and coded values representing different concentrations of each variable. The media were prepared according to these combinations, incl-uding the same concentration of laboratory-grade minerals and buffering agents. The initial pH values of all media were adjusted to 6.5 ± 0.05 and then sterilized at 121°C for 20 min. Each 10 mL of the media formulation was inoculated with 1% (100 μL) of the cell suspension (~10^8^ CFU/mL) and incubated at 37°C for 24 h under static conditions. The response, measured as the viable cell count, was determined, as described in the “Measurement Parameters” section, using the experiment conducted in triplicate. The medium formulation that significantly improved the viable cell production of L22F (with a confidence level exceeding 95%) was selected for further experimentation.

### Effect of agitation speed on L22F cultivation

The effects of different agitation speeds (0 rpm, 120 rpm, and 200 rpm) were evaluated to identify optimal cultivation conditions for L22F. To conduct this experiment, strain L22F was fermented in a 250 mL flask using a modified medium (MM) with a working volume of 50 mL. The pH of the medium was adjusted to 6.5 ± 0.05 using 1 M NaOH and 1 M HCl and then sterilized at 121°C for 20 min. After sterilization, 49.5 mL of the medium was inoculated with 1% (0.5 mL) of the inoculum from the L22F cell suspension (~10^8^ CFU/mL). The flasks were incubated in a shaking incubators at 37°C for 24 h, with viable cell counts, pH values, and residual sugars measured every 6 h as described in the “Measurement Parameters” section. The experiment was conducted in triplicate.

### Comparison of cell viability, pH, and residual sugar levels in MM and commercial MRS medium

The fermentation of L22F was conducted separately in a 500 mL flask using both MM and MRS media, maintaining a working volume of 350 mL. The optimal conditions for L22F fermentation, including temperature, pH, and agitation speed, were esta-blished. Both media were sterilized at 121°C for 20 min. After sterilization, 346.5 mL of each medium was inoculated with 1% inoculum (3.5 mL) from an L22F cell suspension (~10^8^ CFU/mL). Fermentation was carried out under identical conditions. The flasks were incubated in shaking incubator at 37°C for 24 h. Cell viability, pH, and residual sugar levels were measured at 3-h intervals as described in the “Measurement Parameters” section. The experiment was conducted in triplicate.

### Batch fermentation of L22F in a 5 L bioreactor

Scale-up fermentation of L22F was performed using an MM in a 5 L bioreactor (B.E. Marubishi Co., Ltd., Japan). In the preparation of the MM, food-grade minerals and buffering agents were substituted, except for ammonium citrate. After medium preparation, the pH was adjusted to 6.5 ± 0.05 using 1 M NaOH or 1 M HCl. The adjusted medium was then transferred to a bioreactor and sterilized at 121°C for 20 min. A 1% inoculum (0.5 mL) of cell suspensions (~10^8^ CFU/mL) was added to 49.5 mL of sterilized MM in a 250 mL flask and incubated at 37°C for 18 h with the optimal agitation speed. After 18 h, the flask culture was used as the inoculum for subsequent bioreactor fermentation. The sterilized MM (3,465 mL) was inoculated with 1% inoculum (35 mL) from the flask culture. The total working volume in the 5-L bioreactor was 3,500 mL (3.5 L). Fermentation was conducted for 24 h at 37°C with an optimal agitation speed. Samples were collected every 3 h using a sterile pipette. The cell viability, pH, and residual sugar levels were then examined as described in the “Measurement Parameters” section. The experiment was repeated 3 times.

### Batch fermentation of L22F in a 50-L fermenter

Another scale-up of the fermentation of L22F was conducted in a 50-L fermenter (B.E. Marubishi Co., Ltd.) using MM. During the preparation of the medium, food-grade minerals and buffering agents were substituted, except for ammonium citrate. Each medium component was accurately weighed and directly mixed with distilled water in the fermenter. The medium pH was adjusted to 6.5 ± 0.05 using 1 M NaOH and 1 M HCl, and the fermenter containing the medium was sterilized at 121°C for 25 min. The inoculum was prepared in a 500 mL flask with a 350 mL working volume, in which 346.5 mL of the sterilized MM was inoculated with 1% (3.5 mL) of the L22F cell suspension (~10^8^ CFU/mL). The flask was incubated at 37°C for 18 h at the optimal agitation speed. After 18 h, the flask culture was used as the inoculum for 50-L fermentation. Approximately 1% (350 mL) of the inoculum from the flask culture was transferred to 34,650 mL of sterilized MM in the fermenter, resulting in a total working volume of 35,000 mL (35 L). Fermentation was performed for 24 h at 37°C using the optimal agitation speed. Samples were collected every 3 h from the sample withdrawal port using sterile technique. Cell viability, pH, and resi-dual sugar levels in the medium were examined as desc-ribed in the “Measurement Parameters” section. The experiment was performed twice.

### Evaluation of the probiotic properties of L22F using stress challenge assays

Stress challenges were performed to evaluate the probiotic characteristics of L22F strains grown in MM and commercial medium (MRS). The cultures were exposed to heat, oxidative stress, bile, and acid challenge after 24 h of incubation in both MRS and MM. For the heat challenge, 2 mL of the L22F culture was placed in a 15 mL Falcon tube (Corning, USA) and submerged in a 60°C water bath for 10 min. A temperature of 60°C is sublethal and a 10-min exposure is sufficient to assess thermal tolerance without complete inactivation, mim-icking short-term heat stress. The oxidative chall-enge involved adding 1.25 mM hydrogen peroxide to 2 mL of the culture and incubating for 1 h at 37°C. The use of 1.25 mM H_2_O_2_ represents physiological oxidative stress, and a 1-h exposure is sufficient to evaluate the strain’s ability to counteract reactive oxygen species. Acid challenge was conducted by centrifuging 2 mL of the L22F culture at 6,000 × *g* for 10 min, suspending the cells in the same growth medium adjusted to pH 2.5 with HCl, and incubating for 1 h at 37°C. The pH value of 2.5 simulates gastric acidity, and a 1-h exposure reflects the typical gastric transit time that may influence probiotic viability. The bile challenge was performed by inoculating 1% of the L22F cell suspension into 2 mL of MRS and MM, each supplemented with 1% (w/v) bile salts (Oxgall, Sigma-Aldrich, Australia), followed by a 24-h incubation at 37°C. The presence of 1% bile salts mimics intestinal conditions, and 24-h exposure is sufficient to evaluate both immediate bile tolerance and prolonged growth under stress, which are essential for intestinal colonization. CFU counts were performed before and after each challenge to calculate the percentage of surviving cells. The survival rate (%) was calculated using the following formula:

Viable cell count after challenge/viable cell count before challenge × 100.

### Metabolomic analysis

#### Sample preparation

L22F cells were grown separately in a 500 mL flask using both MM and MRS media at a working volume of 350 mL. An approximately 1% inoculum (3.5 mL) from an L22F cell suspension (~10^8^ CFU/mL) was inoculated into 346.5 mL of each medium. The flasks were incubated at 37°C for 12 h in shaking incubators at an agitation speed of 200 rpm. After 12 h of incubation, the culture was centrifuged, the cell-free supernatant collected, and 2 mL transferred to Falcon™ tubes and subjected to protein precipitation for 20 min in an ice bath immersion with 4 mL of cold acetonitrile (Sigma-Aldrich, USA) that was previously stored at −20°C. After centrifugation at 13,528 × *g* for 10 min at 10°C, the upper layer of each sample was collected, transferred to Eppendorf tubes, and concentrated using a vacuum concentrator (Eppendorf™, Finland) for 2 h and 30 min at 30°C. The dry samples were stored at −80°C until analysis [28].

#### Ultra-high-performance liquid chromatography coupled with electrospray ionization quadrupole-time-of-flight mass spectrometry (UHPLC-ESI-QTOF-MS) analysis

The instrument platform used for this analysis was a UHPLC-ESI-QTOF-MS (Bruker’s Compact, Bruker Daltonik, Germany). The separation part was performed using a UHPLC system (Elute UHPLC, Bruker, Darmstadt, Germany) with a Bruker Intensity Solo HPLC column (C18 2.1 × 100 mm, 2 μm). The column temperature and autosampler temperature were set to 55°C and 10°C, respectively. Mobile phase A consisted of 100% HPLC-grade water with 0.1% formic acid (FA), and mobile phase B consisted of 100% methanol with 0.1% FA. The flow rate was set at 0.4 mL/min, and the elution gradient was set as – 99.9% A (0.0–2.0 min and 0.25 mL/min), 99.9%–75% A (2.0–10.0 min and 0.4 mL/min), 20% A (10.0–12.0 min and 0.4 mL/min), 10% A (12.0–21.0 min and 0.4 mL/min), 0.1% A (21.0–23.0 min and 0.4 mL/min), and 99.9% A (24.0–26.0 min and 0.4 mL/min). A sample injection volume of 4 μL was applied for the positive ionization polarity mode, and 70% methanol was used as the blank. After the analysis of a group of five samples, two spectra of pure methanol and one quality control (QC) were acquired to minimize cross-contamination and ensure reproducibility.

Mass spectrometry analyses were performed using a Compact ESI-QTOF system (Bruker), and mass spectral signals were collected in positive-ion scanning mode. Sodium formate (HCOONa) containing 2 mM sodium hydroxide, 0.1% formic acid (FA), and 50% isopropanol was directly injected as an external calibrant at a flow rate of 0.05 mL/min. The conditions in the positive ionization polarity mode were – mass range, 50–1300 m/z; capillary voltage, 4500 V; dry temperature, 200°C; nebulizer, 0.5 bar; and dry gas flow, 4 L/min. The different datasets were analyzed by multivariate analysis. Student’s t-test combined with principal component analysis (PCA) and partial least squares discriminant analysis (PLS-DA) was used to evaluate the differential metabolites among the control and sample groups. For analysis software, the online platform of MetaboAnalyst 5.0 (https://www.metaboanalyst.ca) was used.

### Statistical analysis

Statistical analyses were performed using Minitab version 20 (Minitab LLC, USA) and SAS version 9 (SAS Institute Inc., USA). PBD and RSM analyses were conducted using Minitab to identify and optimize significant factors influencing cell growth. One-way analysis of variance (ANOVA) was used to evaluate the effects of different factors on pH, residual sugar concentration, and viable cell counts. Where significant differences were found (p < 0.05), Duncan’s Mul-tiple Range Test was applied to compare mean values and identify statistically distinct groups. The mean values ± standard deviation (SD) were used to display data from three independent replicates. The SD was used to describe variability among replicates.

## RESULTS AND DISCUSSION

### Carbohydrate utilization pattern of L22F

Understanding the carbohydrate utilization patterns of specific strains is important for selecting the most suitable carbon sources in modified media for industrial purposes. The carbohydrate utilization pattern of strain L22F, as analyzed by the Automated VITEK^®^ 2 Compact system, is shown in Table 1. Strain L22F can utilize D-galactose, D-glucose, D-maltose, sucrose, D-trehalose, D-sorbitol, and D-ribose. D-glucose (dextrose) is a monosaccharide commonly used in most commercial media because of its readily metabolizable form. Sucrose, a readily available disaccharide, can be supplied from food-grade sources. In Thailand, glucose, dextrose monohydrate, and sucrose are rea-dily available and are more cost-effective than other carbo-hydrates [29]. Therefore, an investigation was made of the impact of different concentrations of these selected carbon sources, i.e., glucose, dextrose, and sucrose, on the cell growth of L22F.

**Table 1:** Carbohydrate use pattern of *Lactobacillus plantarum* 22F.

Carbohydrate	Use
D-galactose	+
D-glucose	+
D-maltose	+
D-mannitol	−
D-melezitose	−
Sucrose	+
D-trehalose	+
D-sorbitol	+
D-malate	−
D-ribose	+
Maltotriose	−
D-xylose	−

+=This carbohydrate can be used in L22F, −=This carbohydrate cannot be consumed by L22F, L22F=*Lactiplantibacillus plantarum* 22F

### Screening of carbon sources

The OVAT method was used to screen carbon sources to determine the most influential factors significantly affecting cell production by L22F. Table 2 shows the effect of different carbon sources and their concentrations on the viable cell production of L22F after 24 h of incubation. The viable cell counts at glucose concentrations of 10, 20, and 30 g/L were 8.11 ± 0.01, 8.0 ± 0.03, and 8.05 ± 0.05 log CFU/mL, respectively. For sucrose, the cell counts were 7.77 ± 0.02, 7.78 ± 0.15, and 7.58 ± 0.13 log CFU/mL, whereas for dextrose, they were 7.58 ± 0.17, 7.51 ± 0.24, and 7.53 ± 0.21 log CFU/mL at the same concentrations. Glucose emerged as the most effective carbon source, followed by sucrose and dextrose. This result highlighted the significant impact of different carbon sources on the cell production of L22F, with clear preferences. A previous study by Han *et al*. [30] has reported that glucose is the most effective carbon source for biomass and bacteriocin production in *L. plantarum* YJG. In addition, *L. plantarum* showed a high growth rate in MRS media supplemented with glucose as the carbon source [31]. This preference for glucose is consistent with a study by Ahansaz *et al*. [32] indicating that *L. plantarum* tends to utilize glucose efficiently due to its well-adapted metabolic pathways for hexose sugars, which are crucial for its growth and energy production. In an another study by Kassas *et al*. [33], a low glucose concentration of 11.5 g/L (1.15%) enhanced the growth of *L. plantarum* BH14, likely because higher glucose levels may inhibit cell growth. The required concentration may vary depending on the strain. In the current experiment, glucose at a concentration of 10 g/L was identified as the most effective carbon source, achieving the highest viable cell count. Interestingly, increasing the glucose concentration to 30 g/L did not result in a proportional increase in cell count, suggesting that 10 g/L is the optimal concentration for this strain. Therefore, glucose (10 g/L) was selected as the basic carbon source and concentration for further experiments.

**Table 2:** The effect of different carbon sources and their concentrations on viable cell production of L22F after 24 h of incubation.

Carbon source	Concentration (g/L)	Cell count (log CFU/mL)
Glucose	10	8.11 ± 0.01^a^
	20	8.00 ± 0.03^ab^
	30	8.05 ± 0.05^a^
Dextrose	10	7.58 ± 0.17^cd^
	20	7.51 ± 0.24^d^
	30	7.53 ± 0.21^cd^
Sucrose	10	7.77 ± 0.02^bcd^
	20	7.78 ± 0.15^bc^
	30	7.58 ± 0.13^cd^

Cell count data are presented as the mean ± standard deviation. n = 3. ^a-d^Values with different superscript letters differ significantly (p < 0.05). CFU=Colony-forming units, L22F=*Lactiplantibacillus plantarum* 22F

### Screening of nitrogen sources

**Figure 1:**
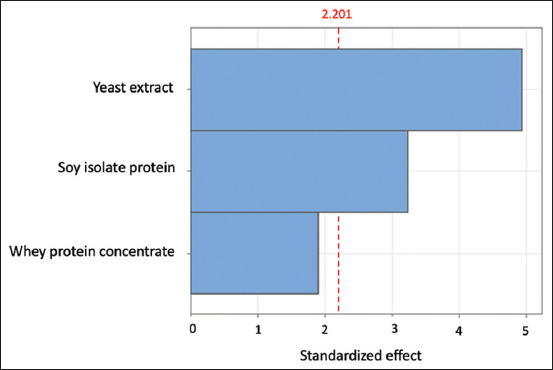
Pareto chart showing the standardized effects of three nitrogen sources on viable cell production of L22F. L22F=*Lactiplantibacillus plantarum* 22F.

The Plackett–Burman experimental design and the response (cell count) of L22F after 24 h of incubation are detailed in Table 3, with viable cell production ranging from 4.98 ± 0.02 to 9.29 ± 0.02 log CFU/mL. Experimental runs with the highest concentrations of SIP and yeast extract gave high cell numbers (>9.2 log CFU/mL). Supplementation with nitrogen sources revealed significant variations in their impact on the cell production of L22F. The viable cell count was increased compared with that of previous carbon sources in a screening experiment, highlighting their crucial role in promoting LAB growth [34]. Figure 1 shows the standardized effects of the three variables as individual bars on the Pareto charts. The bar crossing the vertical (dotted) line indicates statistical significance, indicating that the variable had a notable impact on the cell count. Yeast extract exhibited the greatest effect on the viable cell count of L22F, followed by SIP, whereas WPC had a minimal impact on viable cell production. This indicates that these two sources significantly enhance cell production, likely because of their rich nutrient profiles, which provide essential amino acids, peptides, and vitamins that are critical for bacterial growth and metabolism [35]. The ANOVA results based on the PBD (Supplementary Table S1) demonstrate the effects of nitrogen sources on viable L22F cell production. The impacts of WPC (X1), SIP (X2), and yeast extract (X3) on viable cell production were 0.851, 1.445, and 2.196, respectively. Although all variables positively influenced viable cell production, only SIP (p < 0.001) and yeast extract (p < 0.0001) showed statistically significant effects. Furthermore, the experimental model’s fitness is reflected by the coefficient of determination (R^2^). Typically, R^2^ values range from 0 to 1, with values closer to 1 indicating a better predictive capability. An R^2^ value >0.75 is generally considered acceptable [18]. In this study, the R^2^ value was 0.7952, suggesting that 79.52% of the total variation in the response could be explained (Supplementary Table S1). The superior performance of yeast extract and soy protein isolate can be attributed to their comprehensive nutrient profiles. Yeast extract is particularly known for its high content of B vitamins, free amino acids, and peptides, which are vital for the biosynthetic and energy-yielding pathways of LAB [35]. Similarly, soy protein isolate is a rich source of amino acids and peptides that can be readily utilized by LAB for protein synthesis and other metabolic functions [22, 36]. Consequently, SIP and yeast extract were selected as suitable nitrogen sources for further optimization of the media.

**Table 3:** Plackett–Burman experimental design and response (cell count) to L22F after 24 h of incubation.

Run	X_1_ (g/L)	X_2_ (g/L)	X_3_ (g/L)	Cell count (log CFU/mL)
1	−1 (0)	+1 (30)	−1 (0)	8.75 ± 0.05
2	+1 (30)	+1 (30)	−1 (0)	8.37 ± 0.04
3	−1 (0)	+1 (30)	+1 (30)	9.29 ± 0.04
4	−1 (0)	−1 (0)	−1 (0)	4.98 ± 0.02
5	0 (15)	0 (15)	0 (15)	9.09 ± 0.06
6	+1 (30)	+1 (30)	−1 (0)	8.37 ± 0.04
7	0 (15)	0 (15)	0 (15)	9.05 ± 0.02
8	0 (15)	0 (15)	0 (15)	9.08 ± 0.05
9	+1 (30)	−1 (0)	+1 (30)	9.29 ± 0.02
10	−1 (0)	−1 (0)	−1 (0)	4.98 ± 0.02
11	+1 (30)	+1 (30)	+1 (30)	9.27 ± 0.01
12	+1 (30)	−1 (0)	−1 (0)	6.93 ± 0.06
13	−1 (0)	+1 (30)	+1 (30)	9.26 ± 0.01
14	+1 (30)	−1 (0)	+1 (30)	9.29 ± 0.02
15	−1 (0)	−1 (0)	+1 (30)	9.14 ± 0.01
16	0 (15)	0 (15)	0 (15)	9.11 ± 0.02

X_1_=Whey protein concentrate, X_2_=Soy isolate protein, X_3_=Yeast extract. (+1), highest concentration; (0), central concentration; (−1), lowest concentration. Data are presented as the mean ± standard deviation. n = 3. CFU=Colony-forming units, L22F=*Lactiplantibacillus plantarum* 22F

### Optimization of medium component concentrations using RSM

The RSM using CCD included 20 experimental runs, and the resulting L22F cell counts after 24 h of incubation are detailed in Table 4. The viable cell production ranged from 8.51 ± 0.09 to 9.23 ± 0.04 log CFU/mL. The highest viable cell count, 9.23 ± 0.04 log CFU/mL, was observed in experimental run 15. Experimental run 12 produced a cell count of 9.22 ± 0.05 log CFU/mL, which was not significantly different from that of run 15. Run 12 contained a formulation with 9.0 g/L glucose, 14.1 g/L SIP, and 14.1 g/L yeast extract, making it a cost-effective option at only $2.23 per liter. The model’s accuracy was validated by a statistically insignificant lack of fit (p > 0.05), with a lack of fit value of p = 0.726, confirming the model’s suitability for describing the data (Supplementary Table S2). The RSM highlighted the optimized media formulations for strain L22F and demonstrated the importance of balancing nutrient supply with cost considerations. The use of cost-effective ingredients such as SIP and yeast extract, combined with appropriate concentrations of glucose and essential minerals, ensures high cell viability and robust growth. Consequently, a formulation containing 9 g/L glucose, 14.1 g/L SIP, and 14.1 g/L yeast extract, along with minerals and buffering agents including 1 g/L Tween 80, 2 g/L C_6_H_14_N_2_O_7_, 2 g/L K_2_HPO_4_, 5 g/L CH_3_COONa, 0.1 g/L MgSO_4_·7H_2_O, and 0.05 g/L MnSO_4_·H_2_O, was selected as an alternative MM for the growth of L22F.

**Table 4:** Central composite experimental design and response (cell count) to L22F after 24 h of incubation.

Run	X_1_ (g/L)	X_2_ (g/L)	X_3_ (g/L)	Cell count (log CFU/mL)
1	−α (6.0)	−α (14.1)	−α (14.1)	8.65 ± 0.09^d^
2	0 (7.5)	0 (20.0)	0 (20.0)	8.51 ± 0.09^e^
3	−1 (5.0)	0 (20.0)	0 (20.0)	8.90 ± 0.05^c^
4	α (9.0)	α (26.0)	−α (14.1)	8.52 ± 0.07^de^
5	0 (7.5)	+1 (30.0)	0 (20.0)	9.16 ± 0.06^ab^
6	−α (6.0)	α (26.0)	−α (14.1)	8.52 ± 0.07^de^
7	−α (6.0)	−α (14.1)	α (26.0)	9.19 ± 0.07^ab^
8	α (9.0)	−α (14.1)	α (26.0)	8.87 ± 0.05^c^
9	0 (7.5)	0 (20.0)	0 (20.0)	8.89 ± 0.06^c^
10	0 (7.5)	−1 (10.0)	0 (20.0)	8.90 ± 0.09^c^
11	0 (7.5)	0 (20.0)	0 (20.0)	8.93 ± 0.03^c^
12	α (9.0)	−α (14.1)	−α (14.1)	9.22 ± 0.05^ab^
13	−α (6.0)	α (26.0)	α (26.0)	9.18 ± 0.02^ab^
14	0 (7.5)	0 (20.0)	+1 (30.0)	9.09 ± 0.02^b^
15	+1 (10.0)	0 (20.0)	0 (20.0)	9.23 ± 0.04^a^
16	0 (7.5)	0 (20.0)	0 (20.0)	8.52 ± 0.07^de^
17	0 (7.5)	0 (20.0)	0 (20.0)	8.51 ± 0.09^e^
18	0 (7.5)	0 (20.0)	0 (20.0)	9.13 ± 0.04^ab^
19	0 (7.5)	0 (20.0)	−1 (10.0)	8.52 ± 0.07^de^
20	α (9.0)	α (26.0)	α (26.0)	8.95 ± 0.17^c^

X_1_=Glucose, X_2_=Soy isolate protein, X_3_=Yeast extract. (+1), highest concentration; (−1), lowest concentration; (0), central concentration; (−α, α), axial concentrations. Data are presented as the mean ± standard deviations of three replicates. ^a-e^Values with different superscript letters differ significantly (p < 0.05). CFU=Colony-forming units, L22F=*Lactiplantibacillus plantarum* 22F

### Effect of agitation speed on L22F cultivation

**Figure 2:**
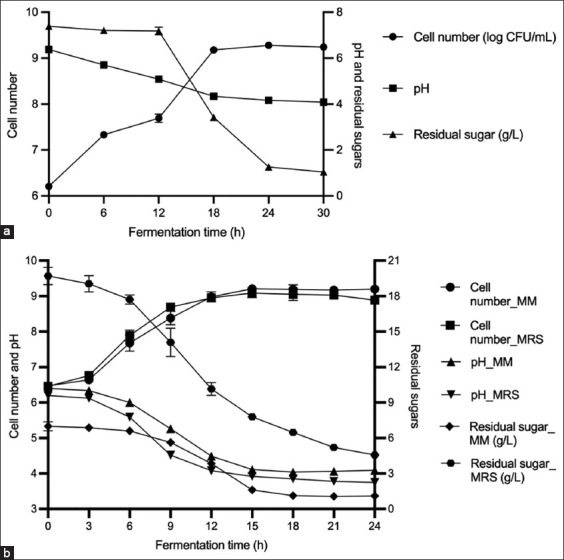
Fermentation of L22F at 37 °C for 24 h with an agitation speed of 200 rpm (a) in a 250 mL flask using modified medium (MM) and (b) in a 500 mL flask using MM compared to de Man, Rogosa, and Sharpe media. Cell numbers, pH values, and residual sugars are shown. L22F=*Lactiplantibacillus plantarum* 22F.

The cell numbers and pH values of L22F cultures grown in 250 mL flasks using the MM at 37°C for 24 h with different agitation speeds are presented in Supplementary Table S3. The highest cell numbers, 9.18 ± 0.02 log CFU/mL, with a pH value of 4.34 ± 0.01, were obtained after 18 h of fermentation at an agitation speed of 200 rpm (Table S3 and Figure 2a). The results provide valuable insights into the optimal conditions for maximizing the growth of L22F. A previous study by Choi *et al*. [18] found that 200 rpm was optimal for maximizing biomass production of *L. plantarum* 200655 in a 5-L bioreactor using a specifically optimized medium, and this is in line with the current study. An agitation speed of 200 rpm ensured adequate oxygen transfer and homogenization of the culture, which are essential for maintaining cellular health and promoting higher cell yields [18, 37]. Effective agitation enhances nutrient availability and waste removal, while also affecting the physiological state of cells, which is essential for achieving high cell densities [38]. Consequently, in further scale-up experiments, an agitation speed of 200 rpm was selected for use.

### Comparison of cell viability, pH, and residual sugar levels in MM and MRS medium

When L22F was grown in a 500 mL flask, the highest cell number (9.21 ± 0.01 log CFU/mL) in the MM was observed at a fermentation time of 15 h, whereas the highest cell number at the same time in MRS media was only 9.09 ± 0.13 log CFU/mL (Figure 2b). The pH values and residual sugar content in the modified and MRS media at 15 h were 4.11 ± 0.05 and 1.62 ± 0.09 g/L and 3.92 ± 0.01 and 7.79 ± 0.08 g/L, respectively (Figure 2b and Supplementary Table S4). These results indicate that the MM not only supported higher cell densities but also more efficient sugar utilization and acid production, which are critical for industrial fermentation processes [37]. The findings suggest that the newly optimized MM is superior to commercial MRS media, enabling higher cell yields, faster fermentation times, and improved substrate utilization and acid production. Consequently, this MM was chosen for further scale-up in 5 L bioreactors and 50 L fermenters, paving the way for more efficient industrial-scale production of the L22F strain.

### Batch fermentation of L22F in a 5 L bioreactor

**Figure 3:**
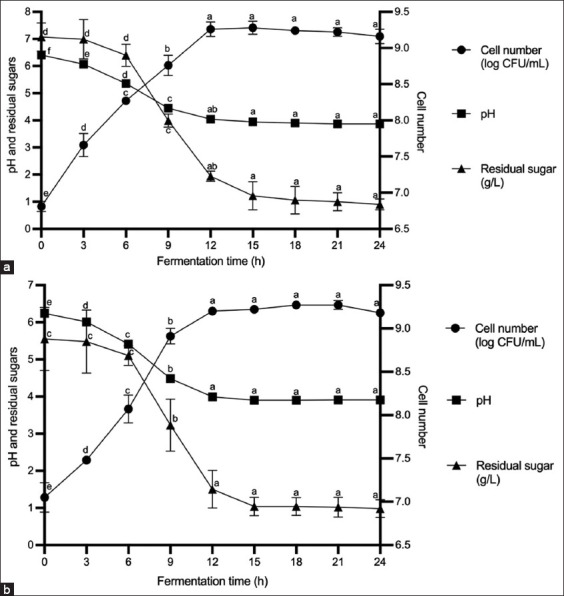
Fermentation of L22F using the modified medium at 37 °C for 24 h with an agitation speed of 200 rpm (a) in a 5 L bioreactor and (b) in a 50 L fermenter. Cell numbers, residual sugars, and pH values are shown. L22F=*Lactiplantibacillus plantarum* 22F.

Batch fermentation of L22F was performed using the modified media in a 5 L bioreactor with a working volume of 3.5 L. As shown in Figure 3a, a logarithmic increase in cell numbers was observed from 0 to 9 h of incubation. Viable cell numbers remained in the stationary phase from 12 h to 21 h, after which they gradually decreased. The highest number of viable cells (9.28 ± 0.09 log CFU/mL) was observed after 15 h of incubation, but this was not significantly higher than the number observed after 12 h (9.26 ± 0.10 log CFU/mL). The pH value of the medium noticeably decreased to 4.04 ± 0.03 at 12 h of fermentation, after which it remained relatively stable until the end of fermentation (24 h). The sugar concentrations in the medium decreased dramatically and reached 1.94 ± 0.19 g/L at 12 h of fermentation; they were almost completely depleted by 24 h of fermentation (Supplementary Table S5).

### Batch fermentation in a 50-L fermenter

Using the modified media, batch fermentation of L22F was carried out in a 50-L fermenter with a working volume of 35 L. A rapid increase in cell numbers was observed from 0 to 9 h of incubation. The number of viable cells was maintained at the stationary phase from 12 h to 21 h and then gradually decreased after 21 h. The highest number of cells (9.27 ± 0.02 log CFU/mL) were observed at 18 h of incubation, but these were not significantly different from the number of cells detected after 12 h (9.20 ± 0.00 log CFU/mL). The medium pH decreased markedly, stabilizing at approximately 3.99 ± 0.01 by 12 h of fermentation, with no signifi-cant changes after that up to 24 h. The level of sugar in the culture medium noticeably decreased and reached 1.50 ± 0.51 g/L at 12 h of fermentation, and it was almost depleted at 24 h of fermentation (Figure 3b and Supplementary Table S5).

Overall, the yields from the modified media were almost the same when scaled up to 5 L and 50 L. The optimal harvest time for L22F was after 12 hours of fermentation, when the maximum number of cells reached 9.20 log CFU/mL (Table 5). The modified media were effective for L22F at different scales, maintaining high cell yie-lds and efficient substrate utilization. These optimal fermentation times will serve as a foundation for future large-scale production, ensuring consistent and cost-efficient probiotic manufacture [29]. The costs per liter of laboratory-grade MRS and food-grade modified media are compared in Supplementary Table S6. The production medium cost of L22F was reduced by approximately 70%–88% compared to commercial MRS media.

**Table 5:** Fermentation of L22F using the modified media in a 5 L bioreactor and a 50 L fermenter, showing cell numbers, residual sugars, and pH values at respective harvest times.

Strain L22F	5L bioreactor	50L fermenter
Harvest time (h)	12	12
Maximum number of cells (log CFU/mL)	9.26	9.20
Residual sugar (g/L)	1.94	1.50
pH	4.04	3.99

CFU=Colony-forming units, L22F=*Lactiplantibacillus plantarum* 22F

### Stress tolerance of L22F grown in MM

**Figure 4:**
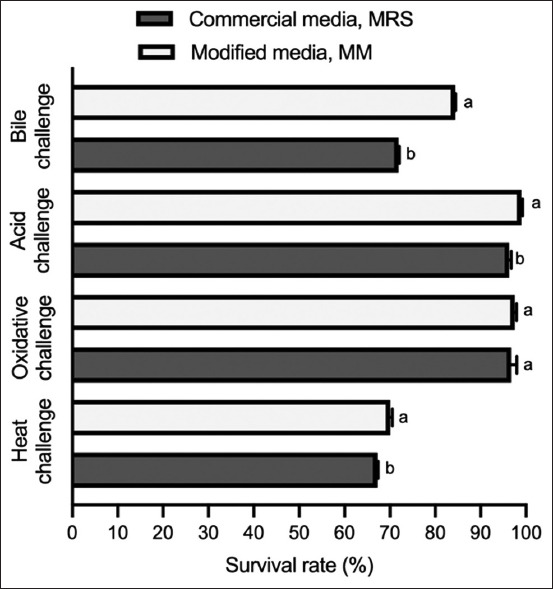
Stress tolerance differences in L22F cultured in the modified medium and commercial de Man, Rogosa, and Sharpe medium. Error bars indicate standard deviations from triplicate experiments. ^a-b^Values with different superscripts differ significantly (p < 0.05). L22F=*Lactiplantibacillus plantarum* 22F.

A probiotic product must be able to tolerate different stressors during manufacturing, storage, transportation, and passage through the gastrointestinal tract. The most common stress factors used in tolerance assays are heat, oxidative stress, bile exposure, and low pH values, representing stages in the processing and digestion of probiotics. L22F was originally selected following favorable acid and bile stress tolerance assays in our previous study by Sirichokchatchawan *et al*. [5]. Zhang *et al*. [39] reported that the majority of amino acids added to the growth medium significantly reduced the tolerance of *Lactobacillus* *rhamnosus* 1301 to heat stress and oxidative stress, indicating that tolerance of *L. rhamnosus* to these stressors can be increased by reducing amino acid intake. Therefore, the researchers recommended that the concentration of nitrogen source components should be optimized to achieve the right balance between biomass and survival of probiotic strains [39]. In our study, L22F cells grown in MM were significantly more tolerant to heat, acid, and bile stress than those grown in MRS, but the oxidative tolerance did not differ between the two conditions (Figure 4). It can be assumed that our modified media contained the right balance of nitrogen sources required for L22F to grow efficiently.

### Metabolomic analysis

#### Determination of whole metabolite profiles

**Figure 5:**
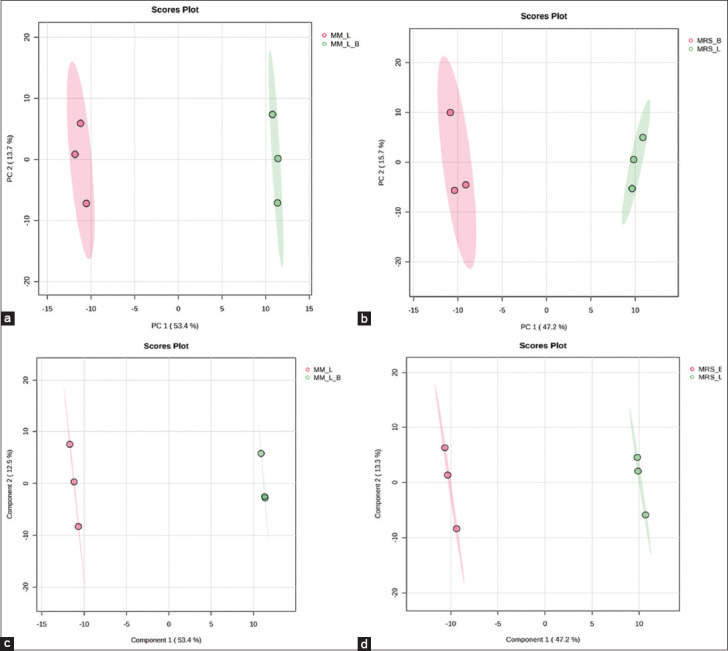
Principle component analysis score plots of modified media before L22F culture, (a) modified medium (MM)_B versus modified media after L22F culture, (b) MM_L, and de Man, Rogosa, and Sharpe (MRS) media before L22F culture, MRS_B versus MRS media after L22F culture, and MRS_L; Partial least squares discriminant analysis score plots of (c) MM_B versus MM_L, and (d) MRS_B versus MRS_L. L22F=*Lactiplantibacillus plantarum* 22F.

Metabolites represent the final products of cell-ular regulatory processes, with their levels regulated in response to environmental changes within biological systems. PCA and PLS-DA are robust statistical modeling techniques for examining inter-sample relationships and distinguishing overall metabolomic differences bet-ween sample groups [40]. In this study, PCA was con-ducted to assess changes in the metabolite profiles of cell-free supernatants before and after L22F culturing in different growth media. Figure 5a illustrates PCA score plots comparing the MM before (MM_B) and after (MM_L) L22F culture, while Figure 5b shows the PCA clustering of the MRS medium before (MRS_B) and after (MRS_L) L22F culture. Both sets of PCA score plots revealed distinct separations between the pre- and post-culture samples, highlighting substantial metabolomic changes induced by L22F culture in both media. We further utilized a supervised PLS-DA model to investigate the specific differences between growth media before and after L22F culture (Figures 5c and 5d), thereby validating the model’s reliability. The classification performance of the PLS-DA model was evaluated using a 5-fold cross-validation method, which included R-squared (R²), Q-squared (Q²), and accuracy values. The closer the R^2^, Q^2^, and accuracy values were to 1, the better the reliability and validation of the model. In our study, all R², Q², and accuracy values were greater than 0.8, demonstrating that the obtained PLS-DA model is reliable and exhibits excellent predictive power. The results showed that the PLS-DA model was valid; thus, the validated variable importance in projection (VIP) values were obtained from this model.

### Identification of differentially abundant metabolites

**Figure 6:**
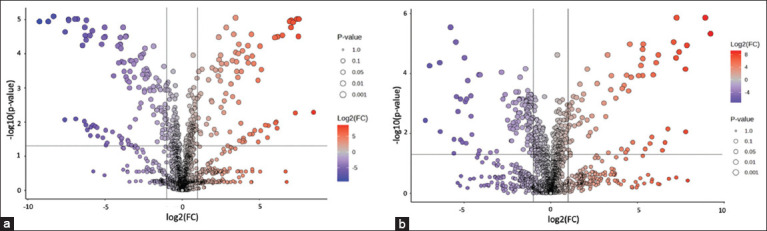
Volcano plot analysis of differential metabolite expression. (a) Modified medium (MM)_B versus MM_L and (b) de Man, Rogosa, and Sharpe (MRS)_B versus MRS_L. The x-axis represents log2 fold change, indicating the magnitude of change for each metabolite, while the y-axis represents −log10 (p-value) derived from the t-test. Metabolites that were significantly upregulated are highlighted in red, those that were significantly downregulated are shown in purple, and metabolites without significant differences are displayed in gray. The size of each dot corresponds to the p-value from the t-test, with larger dots indicating a higher level of statistical significance.

The differentially expressed metabolites were recognized according to the following assessment criterion: p < 0.05 in Student’s t-test and log2 fold change (FC) <−2 or log2 FC >2. Results of the differential analysis are shown in a volcano plot (Figure 6). In the comparison of MM_B versus MM_L, 84 metabolites were significantly upregulated, and 125 were signifi-cantly downregulated following culture (Figure 6a). In MRS_B versus MRS_L, 73 and 126 metabolites were significantly upregulated, and 126 were significantly downregulated (Figure 6b). The VIP values were used to identify the most significantly altered metabolites extracted from the PLS-DA model and to explore their potential biological significance. VIP values >2 were used as the cutoff values for statistical significance. The differential metabolites in MM_B versus MM_L and MRS_B versus MRS_L with VIP values >2 from the PLS-DA model are shown in Supplementary Table S7. A total of 136 metabolites (VIP >2) in MM_B versus MM_L were identified, including 71 from MM_B and 65 from MM_L. The MM for L22F (MM_B) mostly included peptides, followed by nucleic acids, lipids, vitamins, and other compounds. These included valyltryptophan, guanosine, and niacinamide, among others. After 12 h of cultivation, L22F secreted beneficial metabolites into the MM, including indoleacetic acid (ILA), theasaponine, hydroxytyrosol, and delphinidin 3-sophoroside 5-gluc-oside, as shown under MM_L in Table S7. A total of 108 metabolites (VIP >2) were identified in MRS_B versus MRS_L, 58 from MRS_B and 50 from MRS_L. MRS medium for L22F (MRS_B) mostly included peptides, followed by nucleic acids, lipids, vitamins, and other compounds that included valyltryptophan, phen-ylalanyl-alanine, adenine, and riboflavin. After 12 h of cultivation, L22F secreted beneficial metabolites into the MRS medium, including diacetyl, theasaponine, centellasaponin B, and notoginsenoside R10, as shown under MRS_L in Table S7. The metabolomic analysis revealed a notable shift in metabolite profiles as the growth medium changed, and consequently, the bioactive compound production was different. L22F induced the production of suprofen S-oxide, 6-allyl-8β-carboxy-ergoline, 2′,4′-dihydroxy-7-methoxy-8-pre-nyl-flavan, quinidine N-oxide, vicriviroc, lopinavir, and valnemulin exclusively in the MM. In contrast, diacetyl was produced only in the MRS medium. Notably, L22F in the MM (MM_L) produced a higher quantity of significantly upregulated bioactive metabolites with higher abundances compared with the MRS medium.

### Cluster heat map analysis of different metabolites

**Figure 7:**
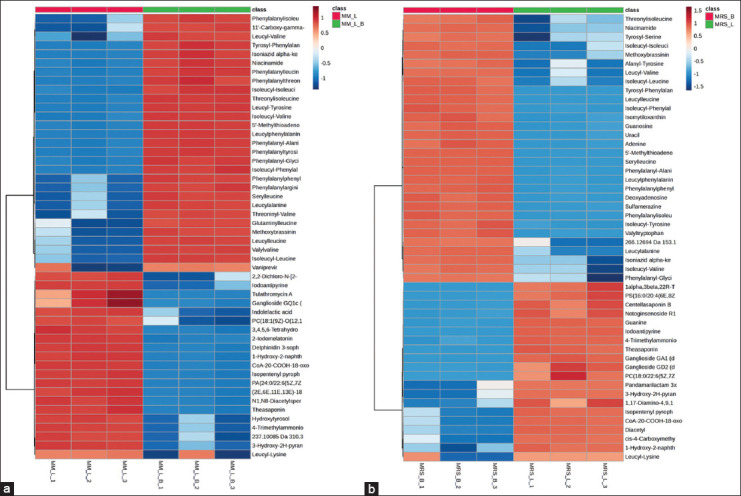
Heat maps of the top 50 metabolites of (a) modified medium (MM)_B versus MM_L and (b) de Man, Rogosa, and Sharpe (MRS)_B versus MRS_L based on variable importance in projection scores >2. Each column corresponds to a sample, each row represents a metabolite, and the color depicts the relative expression level of the metabolite within a group of samples.

Heat maps of the top 50 metabolites of MM_B vs. MM_L and MRS_B versus MRS_L based on VIP scores >2 were drawn to show changes in metabolite concentrations (Figures 7a and 7b). Figure 7a shows that the chemical composition of MM_B was significantly different from that of MM_L. The MM_B group exhibited a high content of dipeptides, including isoleucyl-phenylalanine, isoleucyl-leucine, and phenylalanyl-arginine, which constituted most of its composition, whereas certain vitamin compounds were present in smaller amounts, suggesting that the MM contained a rich source of nitrogen and vitamins to support the growth of L22F. After 12 h of cultivation in the MM, the dipeptides and vitamin compounds identified in the MM_B group were significantly decreased in the MM_L group, suggesting that L22F utilizes these nutrients for its cellular growth. However, the levels of the dipeptides phenylacetylthreonine and leucyl-lysine were signific-antly increased in the MM_L group. This suggests that L22F does not req-uire phenylacetylthreonine or leucyl-lysine as primary nitrogen sources for growth. In addition, the levels of other bioactive metabolites such as ILA, theasap-onine, hydroxytyrosol, and delphinidin 3-sophoroside 5-glucoside were significantly increased in the MM_L group.

Figure 7b clearly illustrates that the chemical composition of the MRS_B medium significantly differed from that of MRS_L. The MRS_B group exhibited a high abundance of dipeptides, including phenylalanyl-alanine, seryl-leucine, leucyl-alanine, and phenylalanyl-glycine, which comprised most of its composition. In addition, it contained smaller amounts of nucleic acids, such as adenine and guanosine, along with some vitamin compounds. These results suggest that the MRS medium provided a rich nitrogen source to support the growth of LAB. After 12 h of cultivating L22F in the MRS medium, the levels of dipeptides from the MRS_B group were significantly decreased in the MRS_L group, except for leucyl-lysine, which was notably upregulated in the MRS_L group. This was also observed in the MM_L group, indicating that L22F does not primarily rely on leucyl-lysine as a nitrogen source for growth. In addition to leucyl-lysine, other bioactive metabolites, including diacetyl, theasaponine, centellasaponin B, and notoginsenoside R10, were significantly increased in the MRS_L group.

### Detection of specific metabolites

**Figure 8:**
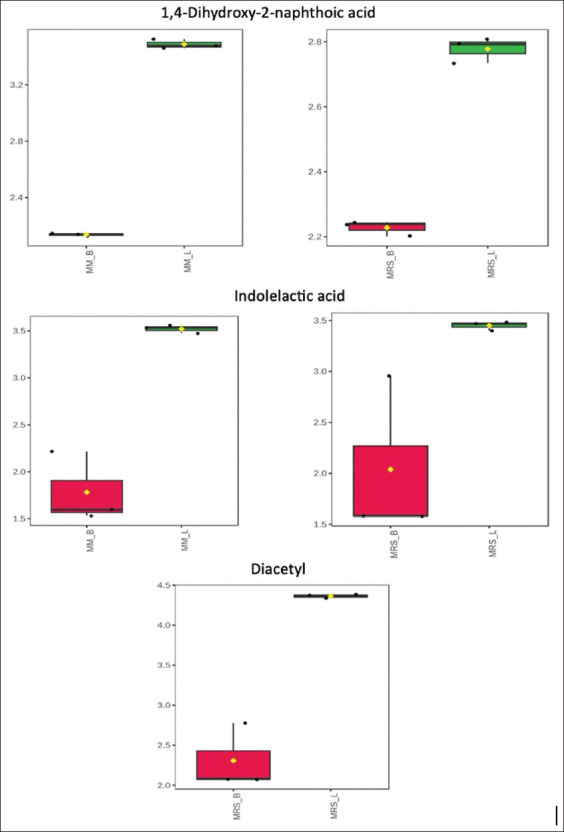
Box plots showing 1,4-dihydroxy-2-naphthoic acid, indoleacetic acid, and diacetyl.

Figure 8 presents box plots illustrating the concen-trations of 1,4-dihydroxy-2-naphthoic acid (DHNA), ILA, and diacetyl. DHNA is a probiotic metabolite derived from bacteria. It is the main component of the metabolic products produced after fermentation by *Propionibacterium freudenreichii* ET-3 [41]. DHNA can stimulate the growth of bifidobacteria, thereby balancing the intestinal bacterial flora [42]. A study by Okada *et al*. [43] reported that DHNA, a novel type of prebiotic, attenuates colonic inflammation not only by balancing the intestinal bacterial flora but also by suppressing lymphocyte infiltration through the reduction of Mucosal addressin cell adhesion molecule-1. In this study, strain L22F produced DHNA in both media. However, a higher abundance of DHNA was observed in our modified media, making it suitable for L22F production. ILA is a molecule produced by gut bacteria that may help improve gut health by reducing inflammation [44], correcting microbial dysbiosis [45], and inhibiting pathogenic bacteria [46]. In the present study, strain L22F produced ILAs in both media. However, a higher abundance of ILAs was observed in our MM, making it an effective medium for L22F production. Diacetyl is a chemical compound used as a flavoring agent in various foods and beverages. It is also known as 2,3-butanedione. It is produced naturally by LAB and is a by-product of fermentation [47]. In our study, diacetyl was produced by L22F only when it was grown in MRS medium. In LAB, diacetyl is produced through the metabolism of citrate to pyruvate, which is then converted to alpha-acetolactate and subsequently decarboxylated to diacetyl. The presence of oxygen and stability of alpha-acetolactate play crucial roles in diacetyl formation. LAB can use citrate in the growth medium. A previous study by Comasio *et al*. [48] reported that the addition of citrate stimulates the production of acetoin and diacetyl in a citrate-positive *Lactobacillus crustorum* strain. Therefore, it is recommended to explore supplementing citrate in our MM for further large-scale production to stimulate diacetyl production for enhancing flavor in the fermented product.

## CONCLUSION

This study successfully developed and validated a cost-effective and scalable fermentation medium for L22F, a swine-derived probiotic strain with demonstrated antimicrobial activity and stress resilience. Among the evaluated components, glucose (10 g/L), soy protein isolate (14.1 g/L), and yeast extract (14.1 g/L) signifi-cantly enhanced cell growth, as confirmed through a systematic optimization approach involving OVAT, PBD, and RSM. The optimized formulation supported high viable cell counts exceeding 9.2 log CFU/mL and maintained consistent performance in 5 L and 50 L fermenters. An agitation speed of 200 rpm was identified as optimal for maximizing biomass production and metabolic activity. Compared to the commercial MRS medium, the modified formulation yielded comparable or superior growth outcomes while reducing production costs by 70%–88%. Stress tolerance assays revealed that L22F cultured in the MM exhibited improved resilience to heat, acid, and bile stress. Metabolomic profiling further indicated that L22F secreted bioactive metabolites such as DHNA and ILA in greater abundance in the MM.

These findings confirm the practical potential of L22F as a functional feed additive for swine production systems. The optimized medium allows for economically viable industrial-scale cultivation while preserving probiotic efficacy, thereby supporting strategies aimed at improving gut health, reducing dependence on antibiotic growth promoters, and enhancing livestock productivity.

A major strength of this work lies in the integration of strain-specific media optimization, use of food-grade and locally available components, and comprehensive functional validation through stress assays and metabolomics. The stepwise optimization approach and consistent scalability further reinforce the reliability and applicability of the developed medium.

Nonetheless, the study has certain limitations. It does not address long-term viability under industrial storage conditions or the *in vivo* effects of L22F administration. In addition, the absence of diacetyl production in the MM indicates a need for formulation enhancement to support flavor compound biosynthesis where desirable.

Future studies should evaluate the *in vivo* efficacy of L22F in swine models, assess its performance under commercial farming conditions, and explore stabilization techniques such as microencapsulation to enhance its stability. Furthermore, modifying the medium composition, particularly by including citrate, could enhance the production of aromatic compounds, such as diacetyl, thereby broadening its functional applications.

In conclusion, this study presents a scientifically robust and economically feasible approach to probiotic production using L22F, offering a promising alternative for sustainable livestock management through targeted microbiome modulation and reduced antibiotic usage.

## DATA AVAILABILITY

The supplementary data can be made available from the corresponding author upon request.

## AUTHORS’ CONTRIBUTIONS

NZM, RK, BK, PP, WS, and NP: Methodology, resources, data curation, and conceptualized the manuscript. NZM, RK, BK, PP, and WS: Software, formal analysis, validated, and investigated the manuscript. NZM and NP: Writing – original draft and visualization. NZM, DJH, and NP: Writing – review and editing. NP: Project administration and supervised the manuscript. NZM, RK, and NP: Funding acquisition. All authors have read and agreed to the publication of the final version of the manuscript.
